# Distant and proximate factors associated with maternal near-miss: a nested case-control study in selected public hospitals of Addis Ababa, Ethiopia

**DOI:** 10.1186/s12905-018-0519-y

**Published:** 2018-01-27

**Authors:** Ewnetu Firdawek Liyew, Alemayehu Worku Yalew, Mesganaw Fantahun Afework, Birgitta Essén

**Affiliations:** 1 Department of Nursing, College of Medicine and Health Sciences, Arba Minch University, Arba Minch, Ethiopia; 2 Department of Preventive Medicine, School of Public Health, Addis Ababa University, Addis Ababa, Ethiopia; 3 Department of Reproductive Health and Health Service Management, School of Public Health, Addis Ababa University, Addis Ababa, Ethiopia; 4 Department of Women’s and Children’s Health, International Maternal and Child Health, Uppsala University, Uppsala, Sweden

**Keywords:** Maternal near-miss, Risk factors, Nested case-control, Public hospitals, Addis Ababa, Ethiopia

## Abstract

**Background:**

Ethiopia is one of the sub-Saharan Africa countries with the highest maternal mortality. Maternal near-misses are more common than deaths and statistically stronger for a comprehensive analysis of the determinants. The study aimed to identify the factors associated with maternal near-miss in selected public hospitals of Addis Ababa, Ethiopia.

**Methods:**

We conducted a nested case-control study in five selected public hospitals of Addis Ababa, Ethiopia from May 1, 2015 to April 30, 2016. Participants were interviewed by well-trained data collectors using pre-tested questionnaire. Medical records were also reviewed to gather relevant information. World Health Organization criteria were used to identify maternal near-miss cases. A total of three controls matched for age and study area was selected for each maternal near-miss case. Bivariate and multivariable conditional logistic regressions were performed using Stata version 13.0.

**Results:**

A total of 216 maternal near-miss cases and 648 controls were included in the study. The main factors associated with maternal near-miss were: history of chronic hypertension (AOR = 10.80,95% CI; 5.16–22.60), rural residency (AOR = 10.60,95% CI;4.59–24.46), history of stillbirth (AOR = 6.03,95% CI;2.09–17.41), no antenatal care attendance (AOR = 5.58,95% CI;1.94–16.07) and history of anemia (AOR = 5.26,95% CI;2.89–9.57).

**Conclusions:**

There is a need for appropriate interventions in order to improve the identified factors. The factors can be modified through a better access to medical and maternity care, scaling up of antenatal care in rural areas, improve in infrastructure to fulfill referral chain from primary level to secondary and tertiary health care levels, and health education to pregnant women.

## Background

In September 2015, the United Nations (UN) General Assembly formally approved a set of 17 Sustainable Development Goals (SDG) as a follow-up to Millennium Development Goals (MDG). Improving maternal health remains an important topic of SDG which is to reduce the global Maternal Mortality Ratio (MMR) to less than 70 per 100,000 live births by 2030 [1]. According to the World Health Organization (WHO), the United Nations Children’s Fund (UNICEF), the United Nations Population Fund (UNFPA), the World Bank Group and the United Nations Population Division (2015) estimate, globally, 303,000 maternal deaths occurred in 2015 with sub-Saharan Africa alone accounts for 66% of the deaths [2].

Ethiopia is one of the sub-Saharan Africa countries with the highest rate of maternal mortality. According to the Ethiopian Demographic and Health Survey (EDHS) report, it was 676 per 100,000 live births in 2011 and the 2016 EDHS documented 412 deaths per 100,000 live births [3, 4]. The government of Ethiopia has made different strategies to lower the rate of maternal mortality. For instance, the members of women’s association of Ethiopia were trained to address social and structural barriers to sexual, reproductive, maternal and newborn health [5]. In addition to this, there was societal level poverty reduction, hospital level allocation of resources, improving skilled birth attendance and reducing home births [5, 6]. Despite all these efforts, there is no significant decline in maternal mortality in the country, where only 27.7% of women received delivery care from skilled provider [3]. Hence, there is a need to assess the possible factors that contributed to maternal mortality. However, maternal deaths are uncommon per each health facility. Thus, in this situation, studies on maternal near-miss could serve as a proxy for maternal death to evaluate quality of obstetric care in particular health institutions [7, 8]. Assessing near-miss has an advantage over maternal death as near-misses are more common and statistically robust [7, 8]. A maternal near-miss is defined by the WHO as ‘a woman who nearly died but survived a complication that occurred during pregnancy, childbirth or within 42 days of termination of pregnancy’ [9].

Different literatures on maternal near-miss around the globe revealed different factors. Advanced maternal age, race, lower socio-economic status, rural residence, less or no antenatal care (ANC) follow-up, multiple pregnancies, nulliparous, multiparous, previous cesarean section delivery, pre-existing medical conditions, overweight and underweight were documented as factors for maternal near-miss [10–18].

The factors associated with maternal near-miss are not well documented in Ethiopia. Few previous published studies conducted in the country relied on patient record to assess predictors of maternal near-miss. Hence, these studies might have limitations of bias due to incompleteness and poor quality of the data at the health facility [19, 20]. However, the current study did not rely on the available hospital secondary data. The aim of this study was to identify the factors associated with maternal near-miss in selected public hospitals of Addis Ababa, Ethiopia.

## Methods

### Study settings and period

We conducted a study in five selected public hospitals of Addis Ababa, capital of Ethiopia from May 1, 2015 to April 30, 2016. The selection of hospitals was based on the number of deliveries conducted per year. In addition, presence of an Intensive Care Unit (ICU), maternity ward, blood transfusion service and availability of cesarean section (CS) delivery were considered in the selection of hospitals. Accordingly, Tikur Anbessa, St. Paul’s Hospital Millennium Medical College, Zewditu Memorial, Yekatit 12 and Gandhi Memorial Hospitals were selected. A total of 29,697 live birth deliveries took place in the participating hospitals during the study period. The details of settings with location map has been described elsewhere [21].

### Study design

A nested case-control study design, matched for age and study setting was employed. Age was categorized in five year interval. Participants were followed from admission till discharge.

### Inclusion criteria of cases

Women who were admitted to the selected hospitals during the study period for treatment of pregnancy-related complications (irrespective of gestational age), who delivered, or were within 42 days of termination of pregnancy, and fulfilled at least one of the conditions that is indicated in the WHO criteria presented in Table 1 [9] were included as cases.

**Table 1:** Identification criteria of maternal near-miss as used by the WHO 2011

Dysfunctional system	Clinical criteria	Laboratory markers	Management based proxies
Cardiovascular	Shock Cardiac arrest	severe hypo perfusion (lactate > 5 m mol/l or > 45 mg/dl) severe acidosis (pH < 7.1)	Use of continuous vasoactive drugs Cardio pulmonary resuscitation
Respiratory	Acute cyanosis Gasping severe tachypnea (respiratory rate > 40 breaths per minute) severe bradypnea (respiratory rate < 6 breaths per minute)	severe hypoxemia (O2 saturation < 90% for ≥60 min or PAO2/FiO2 < 200)	Intubation and ventilation not related to anesthesia
Renal	Oliguria non-responsive to fluids or diuretics	Severe acute azotemia (creatinine ≥300 μmol/ml or ≥3.5 mg/dl)	Dialysis for acute renal failure
Coagulation/hematological	Failure to form clots	severe acute thrombocytopenia (< 50,000 platelets/ml)	Massive transfusion of blood or red cells (≥5 units)
Hepatic	Jaundice in the presence of pre-eclampsia	severe acute hyperbilirubinemia (bilirubin > 100 μmol/l or > 6.0 mg/dl)	
Neurological	Prolonged unconsciousness (lasting ≥12 h)/coma (including metabolic coma), stroke, uncontrollable fits/status epileptics, total paralysis		
Uterine			Uterine hemorrhage or infection leading to hysterectomy

### Exclusion criteria of cases

Those women who have been admitted for reasons not related to pregnancy, delivery or 42 days after termination of pregnancy were excluded.

### Selection of controls

Women who came to the same hospital where the cases happened and, having a similar age- interval category with that of the cases and delivered without any complications were enrolled as a control. For each near-miss case, three controls that occurred within the same day of the near-miss event were included.

### Sample size determination

The sample size was estimated using Epi Info 7 software using sample size determination for unmatched case-control studies. The parameters that were used to estimate the sample size were: confidence level of 95%, power of 80%, case-control ratio of 1:3, expected frequency of exposure in control to be 4.11%, and percent exposure among cases, 10.78%. It was estimated from one study in Ethiopia taking no ANC follow-up as one of the main exposure variable for maternal near-miss that provide the maximum sample size [20]. Accordingly, these yield a minimum sample size of 166 cases and 497 controls. Adding a 10% non-response rate, the final sample size required for the study was 183 cases and 547 controls. To increase the power of the study, all cases observed during one year period (collected for a different objective to determine the incidence of maternal near-miss, which was described elsewhere) [21], along with the corresponding three controls were included in the study.

### Data collection

Women with a maternal near-miss condition and those without any complications during delivery were interviewed by a well-trained midwives and nurses using structured questionnaire. In addition, medical records were reviewed to gather relevant information. Information on socio-economic and demographic characteristics, reproductive health and obstetric history, and pre-existing medical conditions of the women were obtained from the participant’s record. The questionnaires were prepared following a thorough review of literatures. Obstetrics and Gynecology Ward, ICU and Emergency Gynecology Outpatient Department (OPD) of each hospital were visited to collect data. The questionnaires were pre-tested prior to the commencement of data collection to determine the appropriateness of the tool. Data collectors were given a three day training in order ensure consistency of data collection.

### Data analysis

The data were entered using Epi Info 7 software and analyzed using Stata version 13.0. The data were cleaned before analysis. The outcome variable of the study was maternal near-miss. The independent variables which were identified from literatures includes: (i) socio-economic and demographic characteristics (educational level, place of residence, ethnicity, religion, marital status, maternal occupation), (ii) reproductive health and obstetric history of the women (antenatal care booking, parity, history of caesarian section delivery, multiple pregnancies, history of abortion, history of stillbirth, early marriage, female genital cutting) and (iii) pre-existing medical conditions (previous hypertension, previous anemia, human immunodeficiency virus (HIV), history of cardiac problems, history of diabetes mellitus (DM)).

Bivariate logistic regression was performed to examine whether there is a significant association between each individual independent variable and maternal near-miss. For each individual variable, the *P*-value, and unadjusted odds ratio (OR) with its 95% confidence interval, and the number and proportion of each variable of case and control were calculated.

Multivariable conditional logistic regression model was used to examine the independent effect of the factors on the occurrence of maternal near-miss. The variables that were mentioned as factors of maternal near-miss in our literature review were classified as either distant or proximate factors. Socio-economic and demographic variables were taken as a distant factors. Whereas, the rest such as, reproductive health and obstetric history of the women and pre-existing medical conditions were considered as proximate factors. Since distant factors are conceptually related with the proximate factors for the occurrence of maternal near-miss, hierarchical model for the analysis is recommended [22]. Based on this hierarchical order, we have developed two models. All socio-economic and demographic variables with *p* <  0.2 in the bivariate logistic regression analysis were fitted with model 1. Those variables that were significant in model 1 (*p* <  0.05) were fitted with model 2. Model 2 contained those significant variables from model 1 and proximate variables. For each model and variables their adjusted OR, its 95% CI and *P*-value were calculated.

The model fitness was estimated using stata’s fitstat command. Good fit was indicated by a significance value less than 0.05. Both models which were used to determine the factors associated with maternal near-miss were shown to be significant (*p* <  0.0001), which shows the models were best fit.

Multicollinearity among independent variables was assessed by calculating variance inflation factors (VIF). No multicollinearity was suggested during the current analysis as all the calculated VIF were less than 10.

We also defined some of the important independent variables. Educational level was categorized into illiterate (no formal education), primary (grade 1–8), secondary (grade 9–12), and higher education (> 12). Antenatal care visit was considered to be present if a woman reported to have ANC during current pregnancy. Monthly income was categorized into the lowest 25 percentile (below 68 USD), between 25 and 75 percentile (68–181 USD), and above 75 percentile (greater than 181 USD). Marriage before age of 18 was considered as early (based on jurisdiction). Pre-existing medical conditions such as chronic hypertension, anemia, HIV, maternal cardiac disease and DM were considered as present if the women reported their presence before the current pregnancy.

## Results

### Characteristics of the participants

During the one-year period, a total of 238 maternal near-miss cases were reported in all participating hospitals. However, 22 cases were excluded because of incomplete data. Hence, the study included 216 maternal near-miss cases and 648 corresponding controls.

Women with maternal near-miss tended to be illiterate (*P* <  0.0001), never married (*p* = 0.011), reside in rural area (*p* < 0.0001), and had a less monthly income (*p* < 0.0001) compared to controls (Table 2).

**Table 2:** Distribution of selected socio-economic, demographic, reproductive health and obstetric characteristics of women with and without maternal near-miss in Addis Ababa, Ethiopia, 2016

	Near-miss (*n* = 216)	Controls (*n* = 648)	COR (95% CI)	*P-*value
Characteristics	n (%)	n (%)		
Educational level				
Illiterate	61 (30.0)	75 (11.7)	**3.28 (1.85–5.84)**	< 0.0001
Primary	63 (31.0)	214 (33.4)	1.23 (0.70–2.15)	0.470
Secondary	57 (28.1	256 (39.9)	0.91 (0.52–1.57)	0.724
Higher	22 (10.8)	96 (15)	1.00	
Place of residency				
Urban	159 (73.6)	634 (97.8)	1.00	
Rural	57 (26.4)	14 (2.2)	**13.0 (7.12–23.8)**	< 0.0001
Marital status				0.01
Married	200 (92.6)	627 (96.8)	1.00	
Never married	16 (7.4)	21 (3.2)	**2.38 (1.22–4.65**)	0.011
Monthly income				
> 68 USD	81 (37.5)	111 (17.1)	**2.19 (1.43–3.34)**	< 0.0001
68–181 USD	74 (34.3)	370 (57.1)	**0**.**54 (0.36–0.79)**	0.002
> 181 USD	61 (28.2)	167 (25.8)	1.00	
Received ANC				
Yes	183 (84.7)	638 (98.5)	1.00	
No	33 (15.3)	10 (1.5)	**10.8 (5.16–22.6)**	< 0.0001
Number of children				
0–2	171 (79.2)	527 (81.3)	0.99 (0.63–1.56)	0.985
3–4	34 (15.7)	110 (17)	1.00	
> 5	11 (5.1)	11 (1.7)	**3.53 (1.34–9.27)**	0.010
Undergone FGC				
Yes	135 (64.6)	383 (59.6)	0.89 (0.59–1.33)	0.225
No	74 (35.4)	260 (40.4)	1.00	
History of stillbirth				
Yes	21 (9.7)	21 (3.2)	**3.45 (1.79–6.68)**	< 0.0001
No	195 (90.3)	627 (96.8)	1.00	
Early marriage				
Yes	43 (21.5)	90 (14.5)	**1.97 (1.21–3.19)**	0.006
No	157 (78.5)	532 (85.5)	1.00	

Bold data are those which are significant and their significance is indicated by the *P*-values expressed at the right end of each ORs

Compared to the control groups, women with maternal near-miss case were more often did not attend ANC, have greater than five children, have a history of stillbirth and experienced an early marriage, all statistically significant (*p* < 0.05). However, there were no statistically significant difference between cases and controls with regard to presence of previous caesarean section delivery, history of abortion and undergoing a female genital cutting (Table 2).

Cases and controls also differed significantly with regard to the presence of previous medical conditions such as chronic hypertension, anemia, and cardiac problems. However, a significant difference was not observed among the two groups with regard to the presence of HIV and DM (Table 3).

**Table 3:** Distribution of selected previous medical conditions of cases and controls in Addis Ababa, Ethiopia, 2016

	Near-miss (n = 216)	Controls (*n* = 848)	COR (95% CI)	*p-*value
Characteristics	n (%)	n (%)		
Previous hypertension				
Yes	56 (25.9)	16 (2.5)	**13.3 (7.16–24.9)**	< 0.0001
No	160 (74.1)	632 (97.5)	1.00	
Previous anemia				
Yes	73 (33.8)	64 (9.9)	**4.66 (3.12–6.95)**	< 0.0001
No	143 (66.2)	584 (90.1)	1.00	
History of cardiac problem				
Yes	11 (5.1)	5 (0.8)	**6.6 (2.29–18.9)**	< 0.0001
No	205 (94.9)	643 (99.2)	1.00	

Bold data are those which are significant and their significance is indicated by the *P*-values expressed at the right end of each ORs

### Determinants of maternal near-miss

In order to know the factors associated with maternal near-miss, two models were used in a multiple conditional logistic regression analysis. Model one contained five variables which were significant in bivariate analysis (educational level, place of residence, ethnicity, marital status and monthly income). However, the result of the first model showed that only place of residence was found to be associated with maternal near-miss (Table 4). The second model contained eleven variables and five variables remained significant. The factors associated with maternal near-miss in the second model were: history of chronic hypertension (AOR = 10.80,95% CI; 5.16–22.60), rural residency (AOR = 10.60,95% CI;4.59–24.46), history of stillbirth (AOR = 6.03,95% CI;2.09–17.41), no ANC attendance (AOR = 5.58,95% CI;1.94–16.07) and history of anemia (AOR = 5.26,95% CI;2.89–9.57) (Table 5). However, the study did not find that female genital cutting was a determinant factor for maternal near-miss.

**Table 4:** Factors associated with maternal near-miss in model one multiple conditional logistic regression analysis, Addis Ababa, Ethiopia, 2016

	Model 1	
	AOR (95% CI)	*p*-value
Characteristics		
Place of residence		
Rural	**6.86 (3.42–13.76)**	**< 0.0001**
Urban	1	
Educational level		
Illiterate	1.91 (0.95–3.83)	0.068
Primary	1.29 (0.68–2.45)	0.429
Secondary	1.12 (0.62–2.04)	0.699
Higher	1.00	
Ethnicity		
Amhara	1.00	
Oromo	1.32 (0.82–2.13)	0.248
Gurage	0.66 (0.37–1.21)	0.178
Tigre	1.01 (0.41–2.47)	0.975
Silte	0.92 (0.36–2.36)	0.867
Other	0.86 (0.39–1.89)	0.708
Marital status		
Married	1.00	
Never married	1.21 (0.54–2.72)	0.642
Monthly income		
< 68 USD	1.62 (0.95–2.77)	0.075
68–181 USD	0.65 (0.42–1.02)	0.061
> 181 USD	1.00	

Bold data are those which are significant and their significance is indicated by the *P*-values expressed at the right end of each ORs

**Table 5:** Factors associated with maternal near-miss in the last model multiple conditional logistic regression analysis, Addis Ababa, Ethiopia, 2016

	Model 2	
	AOR (95% CI)	*p*-value
Characteristics		
Place of residence		
Rural	**10.60 (4.59–24.46)**	**< 0.0001**
Urban	1	
Received ANC		
Yes	1	
No	**5.58 (1.94–16.07)**	**0.001**
Number of children		
0–2	2.16 (0.09–5.28)	0.09
3–4	1	
> 5	4.27 (0.65–27.98)	0.13
History of still birth		
Yes	**6.03 (2.09–17.41)**	**0.001**
No	1	
Early marriage		
Yes	1.35 (0.66–2.76)	0.411
No	**1**	
Previous hypertension		
Yes	**10.80 (5.16–22.60)**	**< 0.0001**
No	1	
Previous anemia		
Yes	**5.26 (2.89–9.57)**	**< 0.0001**
No	1	
History of cardiac problem		
Yes	3.17 (0.59–16.81)	0.175
No	1	

Bold data are those which are significant and their significance is indicated by the *P*-values expressed at the right end of each ORs

## Discussion

History of chronic hypertension, rural residency, history of stillbirth, no antenatal care attendance and history of anemia were found to be correlated with the occurrence of maternal near-miss.

Among all characteristics, presence of previous chronic hypertension showed the strongest risk factor for the development of maternal near-miss. Women with chronic hypertension are at increased risk for several pregnancy complications which includes: pre-eclampsia, placental abruption, intrauterine growth retardation, CS delivery and pre-term delivery [23]. The finding was consistent with other studies. A study done in Nigeria reported a sevenfold increased risk of maternal near-miss in women with presence of previous chronic hypertension [15]. The observation was also similar to other studies in which the risk of maternal near-miss was higher among women with pre-existing hypertension [24, 25].

Another strong risk factor for maternal near-miss reported in the current study was place of residence. Accordingly, those women who reside in the rural area have higher odds of developing maternal near-miss. A similar finding was also reported in another study in Ethiopia [20]. Studies from Bolivia and Brazil also documented a similar finding [12, 26]. Women from rural area might walk longer to access health services. Particularly when maternal complications occurred, her chance of getting appropriate health care on time might be minimized which in turn increase her chance of morbidity.

Additionally, we found that presence of previous stillbirth in women was an important risk factor for maternal near-miss. After a stillbirth infant, women may experience different psychological as well as relational problems which might in turn increase the risk of maternal complications in subsequent pregnancies. The link between maternal chronic hypertension and stillbirth may also be an alternative explanation [23]. Hence, women who had a stillbirth might have a history of chronic hypertension, and thereby increase the odds of maternal near-miss. Todd et al. in their study on correlates of severe acute maternal morbidity in Kabul also demonstrated that prior stillbirth is a risk factor for maternal near-miss [27].

The study also showed that the odds of maternal near-miss was higher among those women who fail to attend ANC. Different evidences showed that ANC is effective to identify pre-existing factors that could increase the risk of complications during pregnancy or delivery [28, 29]. Protective effect of ANC attendance for maternal near-miss event was also noted in other study too [15]. No ANC attendance could also be associated with some of the identified risk factors of maternal near-miss in the current study such as history of stillbirth and rural residency. However, we have checked the interaction among these variables, and no interaction was noted.

It was also observed that women with a history of anemia have higher odds of maternal near-miss than those without a prior history of anemia. Untreated anemia can lead to post-partum hemorrhage and hypovolemic shock and is a common cause of adverse maternal outcomes [30]. The higher risk of maternal near-miss for women with a prior history of anemia has also been identified in previous studies [14, 31].

Our study did not find that female genital cutting (FGC) was a determinant factor for maternal near-miss events. However, in a WHO multi-center study of female genital cutting, adverse obstetric outcomes were more frequent among cut than uncut women [32]. The possible reason for not getting a significant result in our study might be the fact that the study being underpowered for this specific factor.

This study is the first of its kind in Ethiopia to document the factors associated with maternal near-miss using the newly developed WHO case identification criteria. The use of nested case-control study design had also the advantage of ascertaining cause-effect relationship than a cross-sectional study. The cases and controls were also identified and interviewed prospectively, which helped us to avoid missing important confounding variables. To increase the power of the study, all cases observed during one-year period (collected for a different objective to determine the incidence of maternal near-miss) along with the corresponding three controls were included in the study. Potential sources of biases were also addressed in the current study. For instance, to minimize recall bias, we have taken hospital controls. Hence, the controls were more aware of the antecedent exposure so that there were equivalent degree of recall among cases and controls. In addition, cases and controls were interviewed when they became healthy near to their discharge time. To minimize bias related to measurements, the standardized WHO criteria were used to identify maternal near-miss cases. Furthermore, adequate training was given to data collectors, and there were strict supervision.

As the study was restricted only in public hospitals, it does not represent cases of maternal near-miss happened in private health facilities. The puerperium period defined by the WHO to define maternal near-miss lasts for 42 days post-partum. However, we followed the participants only till hospital discharge. Hence, we were unable to investigate the occurrence of other events such as maternal death occurred after maternal discharge. This might also underestimate the number of maternal near-miss cases reported during the study period.

## Conclusions

History of chronic hypertension, rural residency, prior stillbirth, no antenatal care attendance and presence of prior anemia were the factors independently associated with the occurrence of maternal near-miss. Interventions aimed at improving better access to medical care for pregnant women with a history of chronic hypertension have a paramount importance. Health care professionals need to carefully plan and manage women with prior chronic hypertension. In addition, there is a need for counseling a pregnant woman about the risk of chronic hypertension during routine antenatal care visit. Strengthening the available health system in rural part of the country with focus on maternity service is also a crucial step to avert serious maternal complications. Scaling up of antenatal care in rural areas might have also a role to reduce obstetric risks among pregnant women. An effort to improve in infrastructure could also enhance referral chain from primary level to secondary and tertiary facility-level. Additionally, education of women on the importance of nutrition during pregnancy and supplementation of iron for pregnant women during ANC visits are important steps to avert critical morbidity experience related to anemia. It is also recommended to evaluate the underlying cause of anemia among pregnant women.

## Abbreviations

ANC: antenatal care
AOR: adjusted odds ratio
CI: confidence interval
OR: odds ratio
USD: United States Dollar
VIF: variance inflation factor
WHO: World Health Organization.
